# CAR T cells targeting CD99 as an approach to eradicate T-cell acute lymphoblastic leukemia without normal blood cells toxicity

**DOI:** 10.1186/s13045-021-01178-z

**Published:** 2021-10-09

**Authors:** Jiangzhou Shi, Zijian Zhang, Hong Cen, Han Wu, Shangkun Zhang, Jiaxing Liu, Yingqi Leng, Anqi Ren, Xiyu Liu, Zhijie Zhang, Xiqin Tong, Jinjue Liang, Zhe Li, Fuling Zhou, Liang Huang, You Qin, Kunyu Yang, Tongcun Zhang, Haichuan Zhu

**Affiliations:** 1 Institute of Biology and Medicine, College of Life and Health Sciences, Wuhan University of Science and Technology, Wuhan, 430081 China; 2 Guangxi Medical University Cancer Hospital, Guangxi, 530021 China; 3 Department of Hematology, Zhongnan Hospital of Wuhan University, Wuhan, 430071 China; 4 Department of Hematology, Tongji Hospital, Tongji Medical College, Hua Zhong University of Science and Technology, Wuhan, 430030 China; 5 Cancer Center, Union Hospital, Tongji Medical College, Huazhong University of Science and Technology, Wuhan, 430022 China; 6 Key Lab of Industrial Fermentation Microbiology of the Ministry of Education and Tianjin Key Lab of Industrial Microbiology, College of Biotechnology, Tianjin University of Science and Technology, Tianjin, 300457 China

**Keywords:** CAR T, CD99, T-ALL, AML, Antitumor activity

## Abstract

CAR T cell therapy has shown dramatic clinical success in relapsed or refractory B-ALL and other hematological malignancies. However, the loss of specific antigens, cell fratricide, T cell aplasia, and normal T cell separation are challenges in treating T cell leukemia/lymphoma with CAR T therapy. CD99 is a promising antigen to target T-ALL and AML as it is strongly expressed on the majority of T-ALL and AML. Here, we isolated a low-affinity CD99 (12E7) antibody, which specifically recognizes leukemia cells over normal blood cells. Moreover, T cells transduced with an anti-CD99-specific CAR that contained the 12E7 scFv expanded with minor fratricide and without normal blood cells toxicity. We observed that our anti-CD99 CAR T cells showed robust cytotoxicity specifically against CD99+ T-ALL cell lines and primary tumor cells in vitro and significantly prolonged cell line-derived xenografts (CDXs) or patient-derived xenografts (PDXs) models survival in vivo. Together, our results demonstrate that anti-CD99 CAR T cells could specifically recognize and efficiently eliminate CD99+ leukemia cells.

**Supplementary Information:**

The online version contains supplementary material available at 10.1186/s13045-021-01178-z.

**To the editor:**

T-ALL is an aggressive hematological malignancy accounting for 15% of pediatric and 25% of adult ALL cases [1, 2]. The standard treatment of chemotherapy combined with glucocorticoids has significantly improved survival, but up to 20% of pediatric and 40% of adult T-ALL patients are at risk for relapse [3, 4]. Novel optimal therapeutic strategies need to be developed for T-ALL, particularly for relapsed and refractory T-ALL patients. CARs targeting CD19 have been studied extensively for the treatment of B-ALL [5, 6]. However, CAR T cells share similar antigens with malignant T cells, and translating this approach to T-ALL has been extremely challenging due to fratricide and T cell aplasia [7].

CD99 has been demonstrated to have stronger expression in newly diagnosed T-ALL and also used as a new tool for the detection of MRD [8, 9]. We also confirmed CD99 up-regulated in transcript and protein levels compared with normal T cells (Additional file 1: Fig S1a, b). Further, we found that the CD99 expression is strong in different subgroups with the meanings of FPKM above 100 (Additional file 1: Fig S1c, d), indicating that CAR T cell therapy based on CD99 is a promising therapeutic strategy for T-ALL rooting out. To avoid T cell fratricide and potential on-target, off-tumor effects, we first identified a lower affinity anti-CD99 mAb (12E7) that specifically recognizes CD99-expressing T-ALL cell lines but not normal blood cells (Fig. 1a). Then, we systematically investigated the sensitivity of 12E7 mAb in normal tissue and observed 12E7 mAb-positive signals only in parts of the thymus, but not in the spleen, liver, kidney or other important organs (Additional file 1: Fig S1e). We also found the 12E7 scFv exhibited a lower binding affinity of 6.97 × 10^–8^ M, which is lower than the 1021527 (5.76 × 10^–9^ M) and 3B2/TA8 (1.93 × 10^–9^ M) (Fig. 1b and Additional file 2: Fig S1f). And there was a strong positive correlation between the 12E7 mAb and 12E7 scFv in the different cell lines according to flow cytometry analysis (Fig. 1c). Together, the results indicate that the 12E7 mAb is an optimal antibody for anti-CD99 CAR T therapy according to its specific target molecule recognition and limited binding to normal cells.

**Fig. 1:**
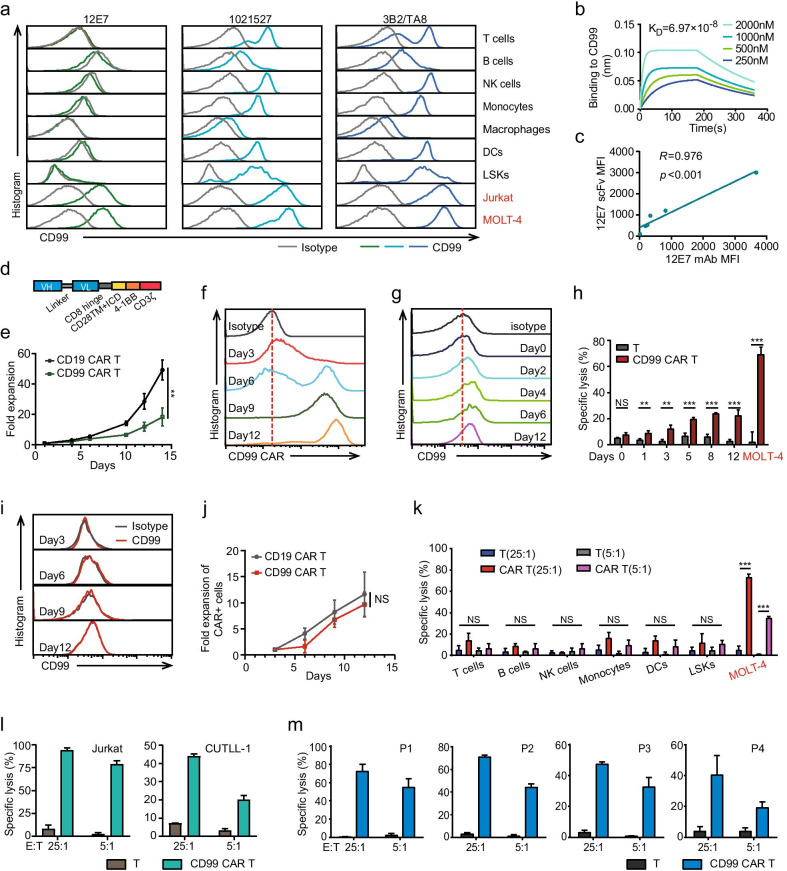
**a** CD99 recognizing ability of three anti-CD99 mAbs (12E7, 1021527 and 3B2/TA8) in normal blood cells and leukemia cell lines (Jurkat and MOLT-4) by flow cytometry. **b** Binding kinetics of anti-CD99 scFv with CD99 protein. Analysis of the interaction between the 12E7 scFv and CD99 protein using BLItz biolayer interferometry. **c** The 12E7 antibody and the anti-CD99 scFv from 12E7 showed a strong correlation in different cell lines based on MFI analysis. **d** Schematic illustration of the anti-CD99 CAR construct. **e** Expansion fold change of total T cells transduced with CD19 CAR or CD99 CAR for 14 days. **f** Percentage of CAR positive cells measured by flow cytometry using Strep-Tag II antibody during the CAR T cells in vitro culture. **g** Expression of CD99 in normal T cells activated by CD3/CD28 beads. **h** Cytotoxic activity of anti-CD99 CAR T cells against normal T cells which activated by CD3/CD28 beads in different days determined by calcein release assay at the ratios of 25:1 after 2-3 h co-culture. **i** Expression of CD99 in CAR positive cells by flow cytometry. **j** Expansion fold change of CAR positive cells for 12 days. **k** In vitro cytotoxic activity of anti-CD99 CAR T cells against different normal blood cells. **l** and **m** Cytotoxic activity of anti-CD99 CAR T cells against T-ALL cell lines (Jurkat/ CUTLL-1) and T-ALL patients’ blasts (Patient #1/ Patient #2/ Patient #3/ Patient #4) as determined by calcein release assay at different E:T ratios (5:1,25:1) after 2-3 h co-culture. IgG as the negative control.***p ≤ 0.001,**p ≤ 0.01,NS = no significant

Next, the 12E7 scFv was incorporated into the lentivirus CAR vector to generate anti-CD99 CAR (Fig. 1d). Following activation and transduction of T cells, anti-CD99 CAR T cells were significantly fewer than the anti-CD19 CAR T control (Fig. 1e). Interestingly, the efficacy of transduced CAR+ cells was significantly increased during cell culture, almost 100% in the 12 days (Fig. 1f). And the following results showed that CD99 induced expression after CD3/CD28 beads activation and could be targeted by anti-CD99 CAR T cells (Fig. 1g, h). In contrast, we purified the CAR+ cells after 3 days transduction and found the antigen of CD99 did not express in anti-CD99 CAR T cells and CAR+ cells could not exhibit significant fratricide during the cell culture (Fig. 1i, j). Next, we assessed the antigen specificity and cytotoxic activity of anti-CD99 CAR T cells in NIH 3T3 human CD99 overexpression cell line and MOLT-4 CD99 knockdown cell line and found that the cytotoxicity was strongly correlated with the expression level of CD99 (Additional file 2: Fig. S1g-h). Furthermore, anti-CD99 CAR T cells showed specifically target the T-ALL cell lines and primary cells but with minimal killing of normal blood cells (Fig. 1k–m and Additional file 2: Fig. S1i). In addition, not only the T-ALL, we also found CD99 expressed and induced powerful antitumor activity in AML and a vast majority of solid tumor cells (Additional file 3: Fig. S1j, k), suggesting that CD99 may be a broad spectrum target for CAR T cell therapy.

To assess the effect of anti-CD99 CAR T cells against CD99+ T-ALL cells in vivo, we performed experiments using CDXs and PDXs models created by Jurkat, MOLT-4, and patient samples, respectively (Fig. 2a). Anti-CD99 CAR T cells conferred robust protection against leukemia progression and significantly extended the median survival of the mice in the CDX and PDX models (Fig. 2b–g). Especially in the PDX-1 model, the engraftment of CD99+ primary T-ALL cells gradually increased over time in the PB of the control T cell groups, whereas anti-CD99 CAR T cells significantly delayed leukemia progression (Fig. 2c). Compared with the T cell treatment group, anti-CD99 CAR T cells significantly eliminated infiltrating leukemia cells in the spleen and BM (Fig. 2h, i and Additional file 4: Fig. S2a-e). More importantly, the results showed that CARs persisted at a detectable level in the PB for at least 21 days in the Jurkat CDX model treated with CAR T cells (Fig. 2j) but without a significant change in animal body weight (Fig. 2k).

**Fig. 2:**
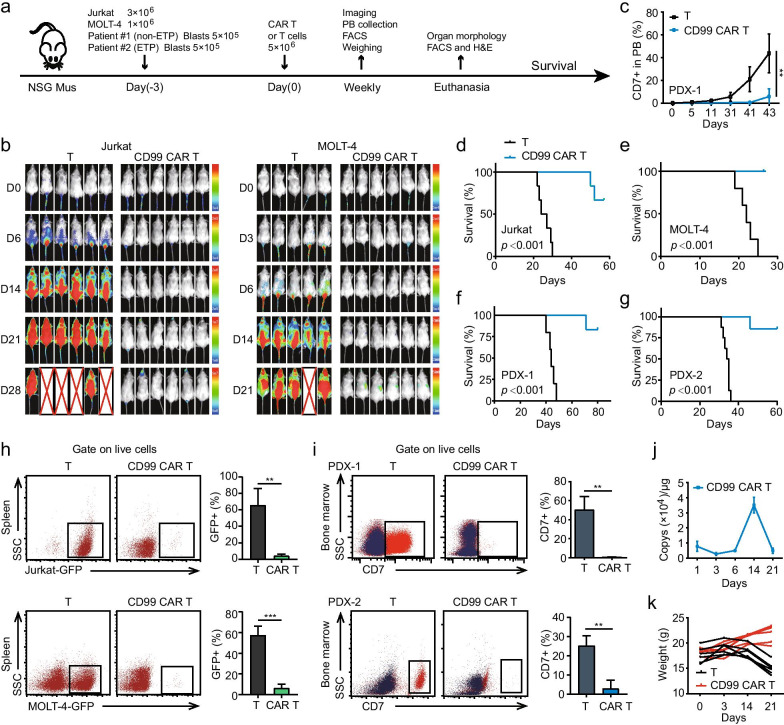
Efficacy and specificity of anti-CD99 CAR T cells in CDXs and PDXs of T-ALL. **a** Schematic outline of the mouse model experiment. NCG mice (n=5/6 per group) were i.v. injected with GFP and luciferase labeled Jurkat, MOLT-4 and two patients’ blast cells (Patient #1 and Patient #2), then, administered 5×106 anti-CD99 CAR T cells or 5×106 T cells per mouse at day 3 following leukemia cells injection. Tumor burden was monitored weekly by IVIS imaging or FACS analysis. All the Methods and Materials were described in the Additional file 6. **b** Tumor progression was monitored using bioluminescent imaging. Scales are normalized for each time points. **c** The proportion of human CD7 positive cells (leukemia cells) in PB of nonETP ALL PDX (derived from patient #1) model from day 0 to day 43. **d**-**g **Kaplan-Meier survival curves of Jurkat CDX mice **d**, MOLT-4 CDX mice **e**, nonETP ALL PDX mice **f** and ETP ALL PDX mice **g** treated with T cells or anti-CD99 CAR T cells. **h** The proportion of GFP positive leukemia cells in the spleen in the T cell or anti-CD99 CAR T cell treatment groups according to FACS analysis. Upper: Jurkat CDX model; Lower: MOLT-4 CDX model. **i** The proportion of human CD7 positive cells in the BM of PDX models. Upper: PDX-1 model; Lower: PDX-2 model. **j** Anti-CD99 CAR T cell expansion and persistence in Jurkat CDX mice. Assessment of the copy numbers of CARs in whole blood cells by q-PCR on different days. **k** The Jurkat CDX mice body weight in different treatment groups. IgG as the negative control. ***p ≤ 0.001, **p ≤ 0.01, NS no significant

In summary, we demonstrated that CD99 is an attractive target for the immunotherapy of T-ALL and provided preclinical evidence for the therapeutic and safe use of fratricide-resistant anti-CD99 CAR T cells. Importantly, future clinical trials will need to assess the safety and feasibility of anti-CD99 CAR T cell therapy.

## Supplementary Information

**Additional file 1: Fig. S1.** (a) Relative CD99 expression was calculated as CD99 fragments per kilobase of exon model per million mapped fragments (FPKM) on T-ALL samples (n=264) and normal PBMC samples (n=23). Data from Dvinge H et al. PNAS, 2014 and Liu Y et al. Nature genetic. 2017. (b) The relative CD99 protein level was calculated as the CD99 mean fluorescence intensity (MFI) on T-ALL samples (n=22) and normal T cell samples (n=5) by flow cytometry. (c) Relative CD99 expression was calculated as FPKM on ETP ALL (n=19), nearETP ALL (n=24) and nonETP ALL (n=146) samples. Data from Liu Y et al. Nature genetic. 2017.(d) Relative CD99 expression was calculated as CD99 FPKM on T-ALL subgroup, including LMO2_LYL(n=18), LMO1/2(n=10), HOXA(n=33), TLX3(n=46), TLX1(n=26), NKX2_1(n=14), TAL1(n=87), TAL2(n=8) and unknown(n=22) samples. Data from Data from Liu Y et al. Nature genetic. 2017. (e) Representative immunohistochemistry (IHC) images of human normal paraffin tissue sections with the CD99 (12E7) mAb. IgG as the negative control.

**Additional file 2: Fig. S1.** (f) Binding kinetics of anti-CD99 antibodies (1021527 and 3B2/TAB) with CD99 protein. Analysis of the interaction between the antibodies and CD99 protein using BLItz biolayer interferometry. (g) The CD99 expression level in NIH 3T3 human CD99-overexpression cell line, and anti-CD99 CAR T cells specifically lysis efficiency at different effector-to-target ratios (1:1/5:1/25:1). (h) The CD99 expression level in MOLT-4 human CD99 knockdown cell line, and anti-CD99 CAR T cells specifically lysis efficiency at effector-to-target ratios (25:1). (i) The gating strategy of blast cells from T-ALL patients and the CD99 expression level in four patients’ blasts (The detail information showed in the Additional file 5: Table S1).

**Additional file 3: Fig. S1.** (j) Upper: Flow cytometry showing CD99 expression in different AML cell lines. Lower: Cytotoxic activity of anti-CD99 CAR T cells against AML cell lines as determined by calcein release assay at different E:T ratios (1:1/5:1/25:1) after 2-3h of co-culture. (k) Upper: Flow cytometry showing CD99 expression in different solid tumour cell lines. Lower: Cytotoxic activity of anti-CD99 CAR T cells against various solid tumour cell lines as determined by calcein release assay at different E:T ratios (1:1/5:1/25:1) after 2-3h of co-culture. ***p ≤ 0.001,**p ≤ 0.01,NS = no significant, Scale bar, 50 μm or 200 μm.

**Additional file 4: Fig. S2.** (a) Spleens from T cell and anti-CD99 CAR T cell treatment groups were weighed and photographed from the PDX-1. (b) The proportion of human CD7 positive cells in the spleen of PDX-1 models. (c) Spleens from T cell and anti-CD99 CAR T cell treatment groups were weighed and photographed from the PDX-2. (d) The proportion of human CD7 positive cells in the spleen of PDX-2 models. (e) Histological features of the spleen in the T cell and anti-CD99 CAR T cell treatment groups (Jurkat, MOLT-4, PDX-1 and PDX-2). Scale bar, 50μm or 200μm.

**Additional file 5**. The patient-related information.

**Additional file 6**. Methods and Materials.

## Data Availability

All supporting data are included in the manuscript and supplemental files. Additional data are available upon reasonable request to the corresponding author.

## Abbreviations

T-ALL: T-cell acute lymphoblastic leukemia
ALL: Acute lymphoblastic leukemia
CAR T: Chimeric antigen receptor T cell
MRD: Minimal residual disease
scFv: Single-chain variable fragment
BM: Bone marrow
AML: Acute myeloid leukemia
CDX: Cell line-derived xenograft
PDX: Patient-derived xenograft
PB: Peripheral blood
