# Supplementary figures and images for: CAR T cells targeting CD99 as an approach to eradicate T-cell acute lymphoblastic leukemia without normal blood cells toxicity
Source: J Hematol Oncol. 2021 Oct 9;14:162. doi: 10.1186/s13045-021-01178-z (PMC8502293; doi:10.1186/s13045-021-01178-z)

Supplementary Fig. 1

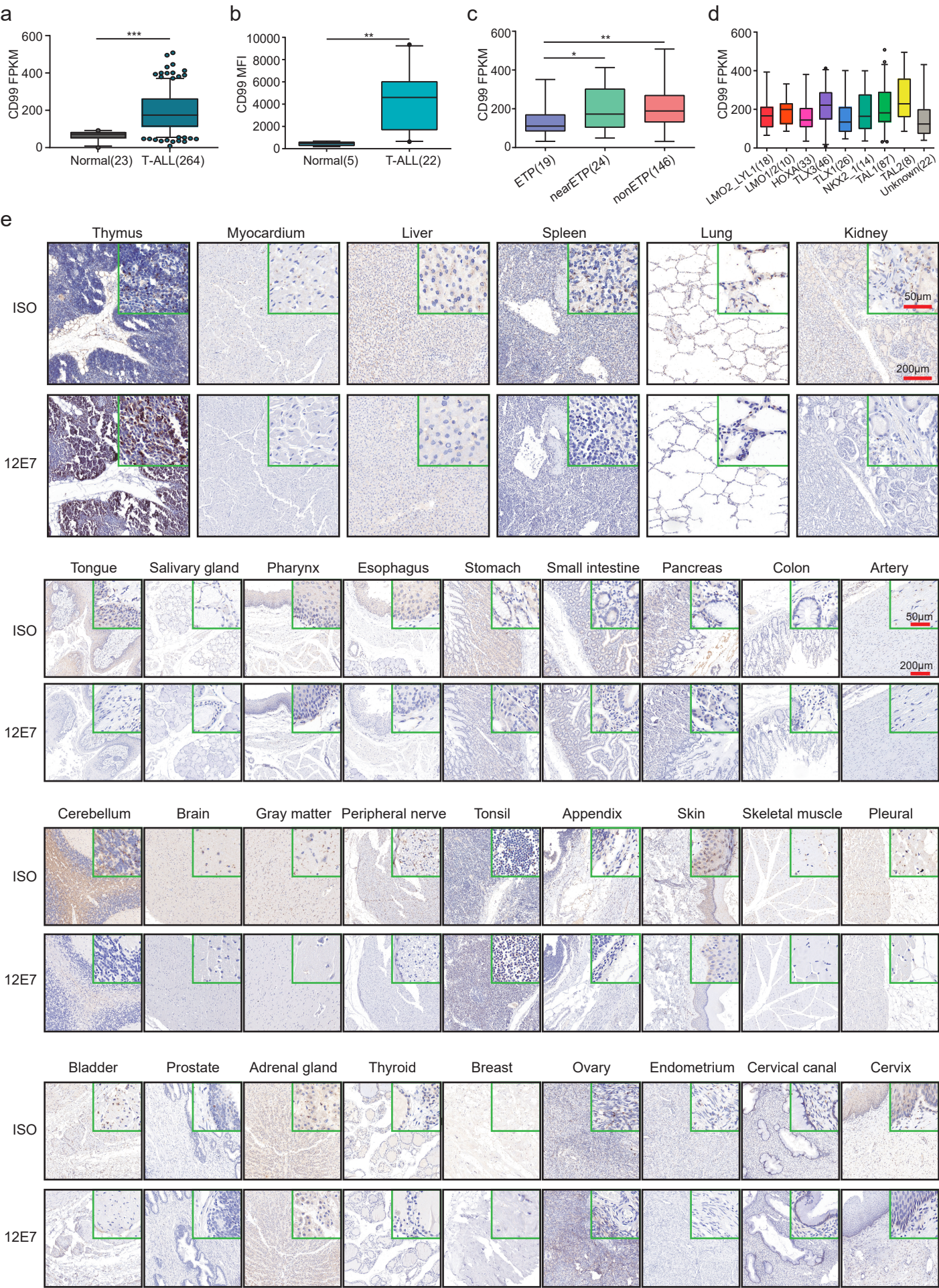

Supplement: Supplementary file 1 — Additional file 1: Fig. S1. (a) Relative CD99 expression was calculated as CD99 fragments per kilobase of exon model per million mapped fragments (FPKM) on T-ALL samples (n=264) and normal PBMC samples (n=23). Data from Dvinge H et al. PNAS, 2014 and Liu Y et al. Nature genetic. 2017. (b) The relative CD99 protein level was calculated as the CD99 mean fluorescence intensity (MFI) on T-ALL samples (n=22) and normal T cell samples (n=5) by flow cytometry. (c) Relative CD99 expression was calculated as FPKM on ETP ALL (n=19), nearETP ALL (n=24) and nonETP ALL (n=146) samples. Data from Liu Y et al. Nature genetic. 2017.(d) Relative CD99 expression was calculated as CD99 FPKM on T-ALL subgroup, including LMO2_LYL(n=18), LMO1/2(n=10), HOXA(n=33), TLX3(n=46), TLX1(n=26), NKX2_1(n=14), TAL1(n=87), TAL2(n=8) and unknown(n=22) samples. Data from Data from Liu Y et al. Nature genetic. 2017. (e) Representative immunohistochemistry (IHC) images of human normal paraffin tissue sections with the CD99 (12E7) mAb. IgG as the negative control. [file 13045_2021_1178_MOESM1_ESM.pdf]

Supplementary Fig. 1

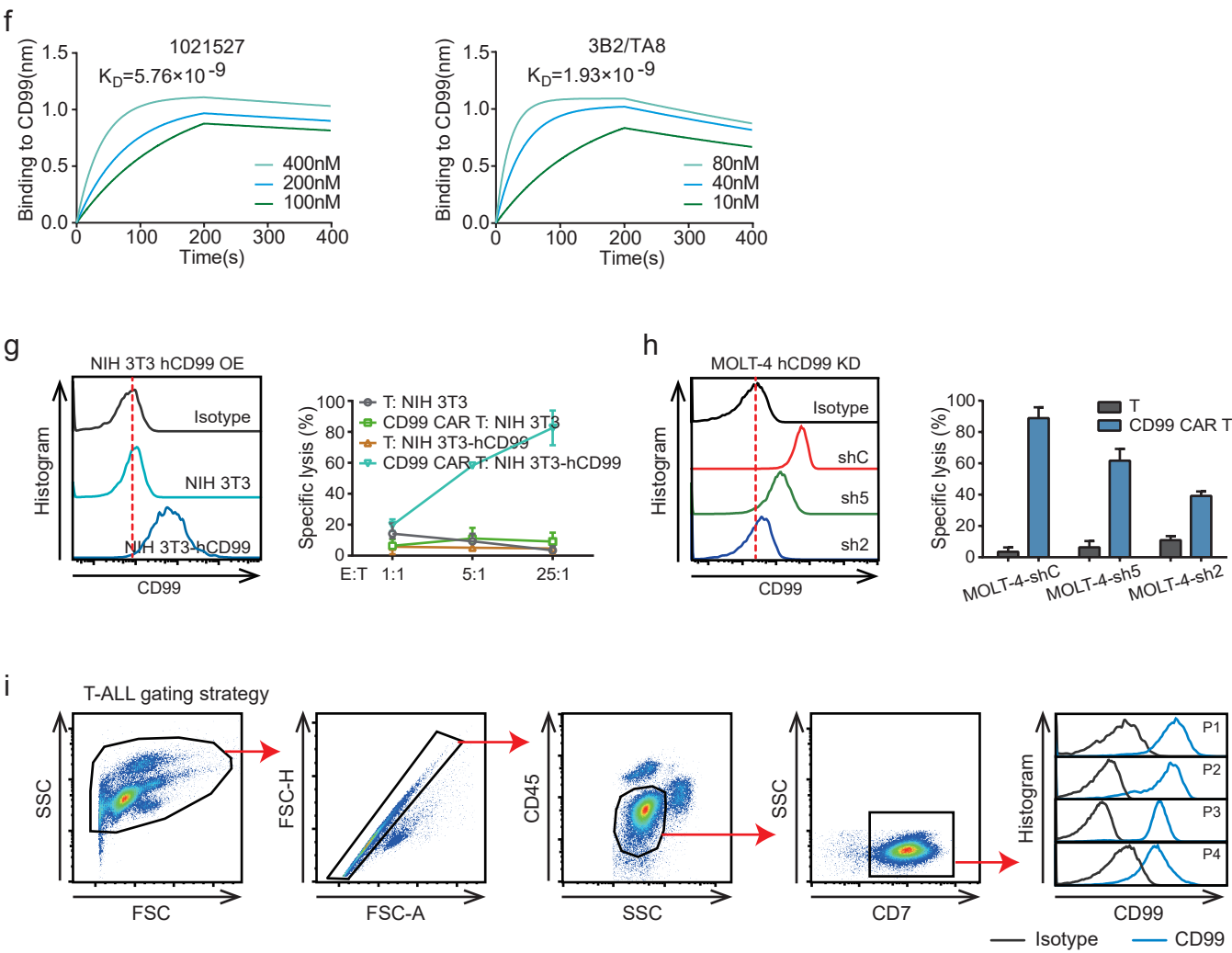

Supplement: Supplementary file 2 — Additional file 2: Fig. S1. (f) Binding kinetics of anti-CD99 antibodies (1021527 and 3B2/TAB) with CD99 protein. Analysis of the interaction between the antibodies and CD99 protein using BLItz biolayer interferometry. (g) The CD99 expression level in NIH 3T3 human CD99-overexpression cell line, and anti-CD99 CAR T cells specifically lysis efficiency at different effector-to-target ratios (1:1/5:1/25:1). (h) The CD99 expression level in MOLT-4 human CD99 knockdown cell line, and anti-CD99 CAR T cells specifically lysis efficiency at effector-to-target ratios (25:1). (i) The gating strategy of blast cells from T-ALL patients and the CD99 expression level in four patients’ blasts (The detail information showed in the Additional file 5: Table S1). [file 13045_2021_1178_MOESM2_ESM.pdf]

Supplementary Fig. 1

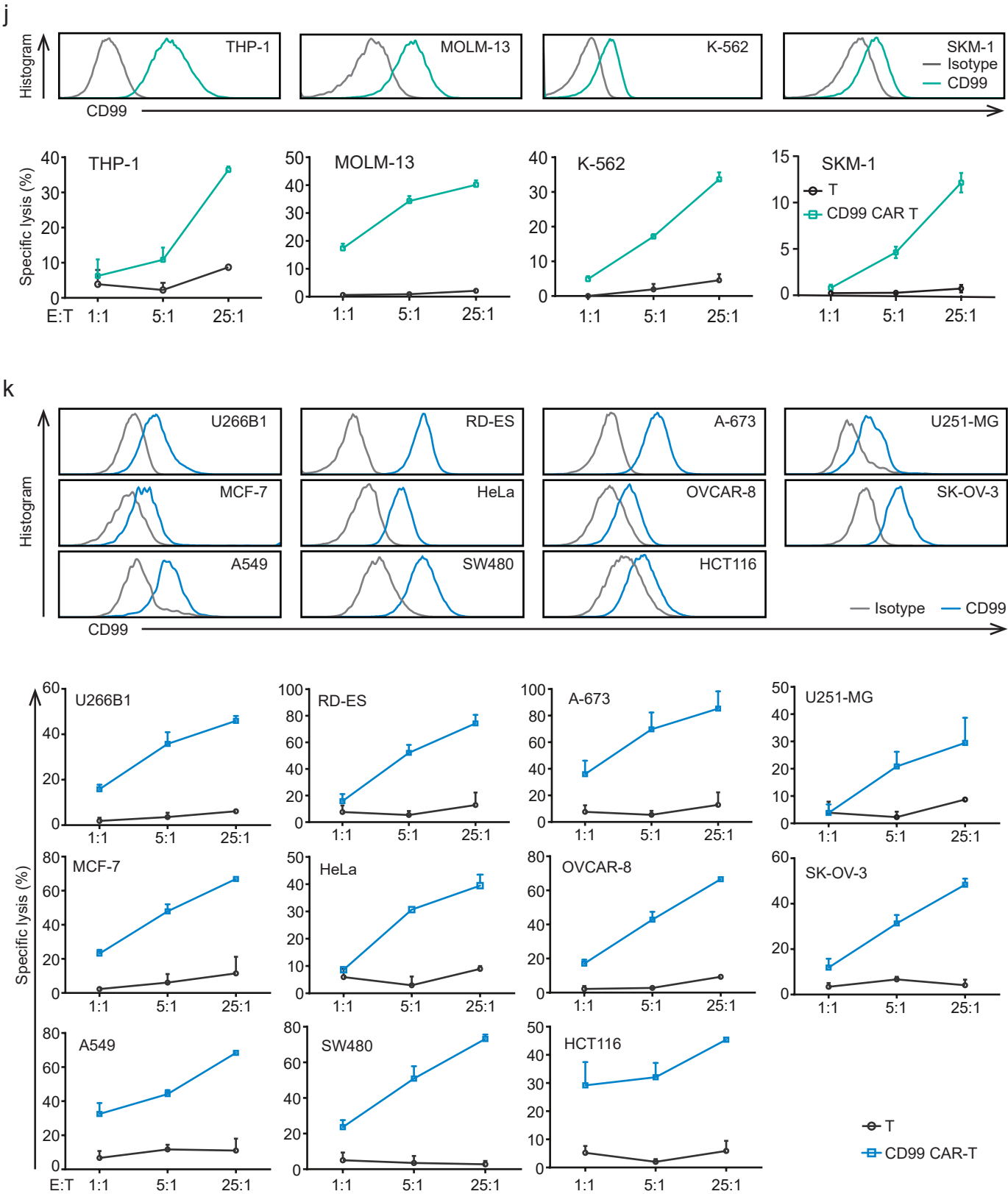

Supplement: Supplementary file 3 — Additional file 3: Fig. S1. (j) Upper: Flow cytometry showing CD99 expression in different AML cell lines. Lower: Cytotoxic activity of anti-CD99 CAR T cells against AML cell lines as determined by calcein release assay at different E:T ratios (1:1/5:1/25:1) after 2-3h of co-culture. (k) Upper: Flow cytometry showing CD99 expression in different solid tumour cell lines. Lower: Cytotoxic activity of anti-CD99 CAR T cells against various solid tumour cell lines as determined by calcein release assay at different E:T ratios (1:1/5:1/25:1) after 2-3h of co-culture. ***p ≤ 0.001,**p ≤ 0.01,NS = no significant, Scale bar, 50 μm or 200 μm. [file 13045_2021_1178_MOESM3_ESM.pdf]

Supplementary Fig. 2

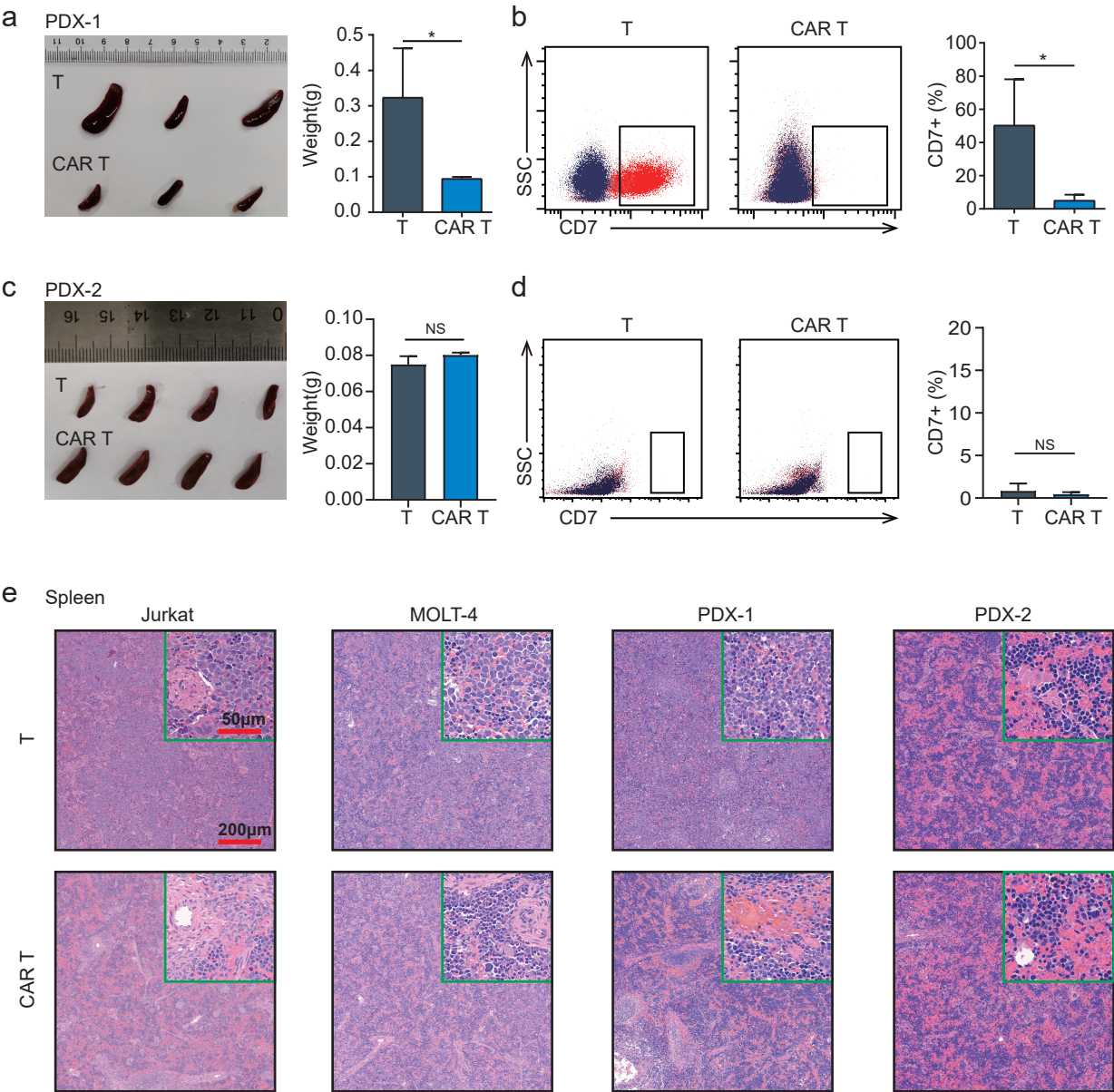

Supplement: Supplementary file 4 — Additional file 4: Fig. S2. (a) Spleens from T cell and anti-CD99 CAR T cell treatment groups were weighed and photographed from the PDX-1. (b) The proportion of human CD7 positive cells in the spleen of PDX-1 models. (c) Spleens from T cell and anti-CD99 CAR T cell treatment groups were weighed and photographed from the PDX-2. (d) The proportion of human CD7 positive cells in the spleen of PDX-2 models. (e) Histological features of the spleen in the T cell and anti-CD99 CAR T cell treatment groups (Jurkat, MOLT-4, PDX-1 and PDX-2). Scale bar, 50μm or 200μm. [file 13045_2021_1178_MOESM4_ESM.pdf]
