# Supplementary material for: CAR T cells targeting CD99 as an approach to eradicate T-cell acute lymphoblastic leukemia without normal blood cells toxicity
Source: J Hematol Oncol. 2021 Oct 9;14:162. doi: 10.1186/s13045-021-01178-z (PMC8502293; doi:10.1186/s13045-021-01178-z)
Supplement: Supplementary file 5 — Additional file 5. The patient-related information. [file 13045_2021_1178_MOESM5_ESM.docx]

| Cases | Gender | Age | Status^1^ | Phenotype of Blasts | Genomics information |
| --- | --- | --- | --- | --- | --- |
| P1 | Male | 47 | nonETP-ALL | Blasts (19.6%) are positive for CD2, CD5, and partially positive for **CD99,** cCD3, CD7, CD4, while negative for CD13, TDT, CD34, CD3, CD8, CD117, CD33, CD1a, MPO, CD79a, CD56. | WT1^+^, BCR/ABL1^+^ |
| P2 | Female | 69 | ETP | Blasts (85.4%) are positive for TDT, **CD99**, CD7, cCD3, CD33, and partially positive for CD19, while negative for CD2, CD5, CD3, CD13, CD11b, CD8, CD11c, CD117, CD10, CD34, HLA-DR, CD4, TCRa/b, TCRr/d, CD11c, CD20, CD22, CD1a, MPO, CD79a, CD56, CD16, CD14, CD64, CD1a, CD15. | WT1^+^ |
| P3 | Male | 15 | ETP | Blasts (90.1%) are positive for CD34, CD33, **CD99**, CD7, CD38, CD117, and partially positive for cCD3, CD13, CD11b, CD11c, while negative for CD2, CD56, CD10, CD5, CD3, TCRa/b, TCR/rd, TDT, HLA-DR, CD8, CD19, CD1a, CD4, MPO, CD79a, CD16, CD14, CD64, CD1a, CD15. | WT1^+^, TCRG rearrangement |
| P4 | Male | 15 | nonETP-ALL | Blasts (91.1%) are positive for CD8, CD5, **CD99**, CD7, CD38, cCD3, CD2, CD3, and partially positive for HLA-DR, TDT, while negative for CD34, CD13, CD11b, CD11c, CD117, CD33, CD19, TCRa/b, TCRr/d, CD10, CD20, CD22, CD1a, CD4, MPO，CD79a, CD56, CD16, CD14, CD64, CD1a, CD15. | WT1^+^, SIL/TAL1^+^, TCRB, TCRG rearrangement |

Table S1

^1^ ETP-ALL is defined by a distinctive phenotype characterized by a lack of expression of CD1a and CD8, weak or absent expression of CD5 and aberrant expression of one or more myeloid or stem cell markers (Coustan-Smith et al., 2009).
