# Supplementary material for: CAR T cells targeting CD99 as an approach to eradicate T-cell acute lymphoblastic leukemia without normal blood cells toxicity
Source: J Hematol Oncol. 2021 Oct 9;14:162. doi: 10.1186/s13045-021-01178-z (PMC8502293; doi:10.1186/s13045-021-01178-z)
Supplement: Supplementary file 6 — Additional file 6. Methods and Materials. [file 13045_2021_1178_MOESM6_ESM.docx]

**Methods**

**Cell lines**

NIH 3T3, MOLT-4, Jurkat, THP-1, K-562, A-673, U251-MG, MCF-7, HeLa, SK-OV-3, A549, and SW480 cell lines were purchased from the Chinese Academy of Sciences Cell Bank (CASCB, China). U266B1, RD-ES, OVCAR-8 and HCT116 cell lines were purchased from the American Type Tissue Culture Collection (ATCC, USA). SKM-1, MOLM-13 and OCI-AML3 were purchased from DSMZ(Germany). The Lenti-X 293T cell line was obtained from TAKARA. All the cells identity was confirmed by STR loci profiling performed by source of institutes.

NIH 3T3, A-673, U251-MG, MCF-7, HeLa, SK-OV-3, A549, OVCAR-8, HCT116 and Lenti-X 293T cell lines were cultured in DMEM with 10% FBS. Jurkat, MOLT-4, THP-1, K-562, U266B1, RD-ES, SW480, SKM-1 and MOLM-13 and cell lines were cultured in RPMI 1640 medium with 10% FBS. Cell lines were placed in a 37°C and 5% CO2 humidified incubator and tested to ensure the lack of mycoplasma contamination.

**Biolayer interferometry (BLI)**

To analyse the binding kinetics of the anti-CD99 scFv or antibodies with CD99 protein, we performed biolayer interferometry on an Octet QK system against anti-CD99 scFv. The biosensor was coated with streptavidin, and the CD99 protein was biotinylated. Then, the CD99 protein was immobilized onto the biosensor by biotin-streptavidin interactions. After removal of excess biotin, anti-CD99 scFv or antibodies was injected over the surface of the biosensor for association. Then, ForteBio BLItz was used to assess the binding affinities following the protocol. All BLI experiments were repeated at least three times, and the affinities of CD99 and anti-CD99 scFv or antibody were determined by binding curves using GraphPad Prism.

**CAR construction and lentivirus production**

The CAR backbone consists of a CD8 hinge, a transmembrane domain, a CD28 intracellular costimulatory domain, a 4-1BB costimulatory domain and a CD3ζ activation domain, as illustrated in Figure1d. The sequence of 12E7 scFv come from previous patent (patent number: WO2015/161267 A2). The codon-optimized CD99 CAR was synthesized (Sangon Biotech; China) and cloned into the lentiviral vector PTK. To produce CD99-CAR lentivirus, 293T cells were transfected with a combination of plasmids containing PTK-anti-CD99 CAR, pMDLg-pRRE, pRSV-rev and pMD2.G.

**CAR T cell manufacturing**

The peripheral blood mononuclear cells (PBMCs) from the volunteers were isolated through Ficoll-Hypaque density gradient centrifugation (GE Healthcare, Sweden), and T lymphocytes were positively selected from PBMCs using MACS® human CD3 microbeads (MACS, Germany) following the manufacturer’s instructions. T cells were activated with Transact (MACS, USA) for 1 day and then transduced with the above lentivirus at a multiplicity of infection (MOI) ranging from 3 to 5. To determine the transduction efficiency of anti-CD99 CAR, cells were stained with Strep-Tag II antibody and analyzed by flow cytometry after 3 days. Throughout manufacturing, all CD3+ T cells were cultured using GMP medium (TexMACS™, USA) with additional IL-2 (Miltenyi, Germany), HEPES (InvivoGen, USA) and L-glutamine (Gibco, USA). The viability, potency, copy number, replication-competent lentivirus, sterility, mycoplasma, and of CAR T cells were analyzed for quality control purposes.

**Enrichment of CAR positive T cells**

CAR T cells were enriched using anti-FITC MicroBeads (Miltenyi Biotec) according to the manufacturer's instructions. Briefly, the CAR T cells were labeled with FITC-conjugated antibody (Strep-Tag II-FITC) and anti-FITC MicroBeads, and then the labeled cells were put into a magnetic field. Using this procedure, the magnetically labeled FITC-positive cells were retained in the tube.

**Flow cytometry**

All reagents and antibodies for flow cytometry analysis were purchased from Biolegend / BD / R&D unless stated otherwise. Flow cytometry was performed using a Beckman CytoFLEX flow cytometer. FACS sorting was performed using a BD FACSAria (BD Biosciences, USA). Cell lines were incubated with antibodies at the recommended dilution on ice for 30 mins in the dark and then washed twice with PBS containing 2% FBS (Gibco, USA). PB were lysed with red blood cell (RBC) lysis buffer to remove the red blood cells to obtain mononuclear cell suspensions and prepared through the process described above. Cells were subsequently stained with 7-AAD to exclude dead cells. Positive events were determined by isotype control gating for each antibody. The efficiency of anti-CD99 CAR transduction was calculated using Strep-Tag II. Information pertaining to all antibodies used in this study is shown in Antibody Table showed below. Data analysis was carried out using FlowJo (v10.0.7, FlowJo, LLC, Ashland, OR, USA).

**Knockdown and Overexpression**

For antigen specificity analysis of anti-CD99 CAR T cells, knockdown or overexpression cell lines were generated as follows. To establish short hairpin (sh) CD99 knockdown cell lines, MOLT-4 cells were infected with lentivirus packaged by the silencing plasmid (PLVX-H1-EF1a-GFP-IRES-puro), which includes a puromycin resistance region, and an shRNA construct to target CD99 (shRNA2: 5’- CGGATGGTGGTTTCGATTTATCTCGAGATAAATCGAAACCACCATCCGTTTTTT-3’; shRNA5: 5’-CCAGCTGTTCAGCGTACTCTTCTCGAGAAGAGTACGCTGAACAGCTGGTTTTTT-3’) or a non-specific shRNA control (shRNA C: 5’- CAACACAGATGATAGAGCACCAATTGGTGCTCTATCATCTGTGTTGTTTTT-3’). MOLT- 4 cells stably expressing CD99-targeted shRNA (MOLT-4 sh2 and MOLT-4 sh5) and nonspecific shRNA control (MOLT-4 shC) were analyzed by flow cytometry to measure the knockdown efficiency. To construct a stable cell line overexpressing human CD99, a Lentivirus vector containing the CDS of CD99 was transfected into NIH 3T3 cell lines. The overexpression efficiency of NIH 3T3-hCD99 was measured by flow cytometry as described above.

**Cytotoxicity assay**

The cytotoxicity of CAR T cells was measured by the calcein release assay with all assays performed in triplicate at least. Target cells were labelled with 25 µM calcein-AM (Aladdin, China) at 37°C for 30 mins, washed three times and then co-cultured with effector cells at the Effector: Target cells ratios of 25:1, 5:1, and 1:1 in 96-well plates. After 2.5h of co-incubation, the supernatant was harvested, and the fluorescence intensity (FI) of the calcein released into the media was quantified using a microplate reader (PerkinElmer Victor X3, USA). The tumour killing efficiency was calculated as previously reported (Liu J et al. J Hematol Oncol. 2021).

**Mouse models**

Mouse experiments were performed with equal numbers of 6-to 8-week-old NOD/SCID/IL-2Rγ-null (NCG) mice (Jiangsu GemPharmatech Co, Ltd,China) with the approval of the Animal Ethics Committee of Wuhan University of Science and Technology of ID WKD-Zhu-1.

To establish CDX mouse models, mice were sub-lethally irradiated and intravenously injected with 3x10^6^ Jurkat cells or 1x10^6^ MOLT-4 cells. The T-ALL cell lines used were transduced with pLVX-EF1α-Luciferase-GFP lentiviral vectors. Following engraftment, The luciferase signals by bioluminescent imaging on day 3 were distributed evenly across the groups: one group received 5x10^6^ anti-CD99 CAR T cells, and one group received 5x10^6^ non-transduced T cells. Imaging of mice was acquired with a Xenogen IVIS 200 instrument (Perkin Elmer, USA) weekly and analyzed using Living Image software (PerkinElmer, USA). The PB of animals was assessed every 7 days by flow cytometry. Studies were terminated by euthanasia of mice when total flux reached 10^9^(p/s/cm^2^sr), and the spleens were processed for weight measurement and IHC staining.

For the PDX mouse models, briefly, the mice were sub-lethally irradiated and intravenously injected with 5x10^5^ fresh T-ALL Blast cells from patients(patient #1 and patient #2). After 3 days, the mice were randomized into groups and intravenously injected with 5x10^6^ anti-CD99 CAR T cells or 5x10^6^ non-transduced T cells. We also collected PB at different stages of tumor progression, monitored the CAR copy number at each time point and performed other experiments as described.

**Quantitation of CAR in mouse models**

The copy number of anti-CD99 CAR T was analyzed by quantitative PCR. Genomic DNA from CAR T cells was isolated using a DNA Isolation kit and diluted to a concentration of 50 ng/uL, according to the manufacturer’s instructions. q-PCR was carried out with primers and fluorescent probe: anti-CD99-F:CTTCGCAGCCTATCGCTCCAA anti-CD99-R:ACTTCACTCTCAGTTCACATCCTCC and anti-CD99-probe: Fam-TCGGCAGCTACAGCCATCTTCCTCTTGAGTAGT-Tamra. The anti-CD99 CAR genome copy number was normalized to the standard linear equation, which was obtained from serially diluted Anti-CD99 CAR plasmid standard DNA (from 10^1^-10^8^ fold series). The results were reported as copies of the CAR genome per µg genomic DNA.

**Histopathology and immunohistochemistry (IHC)**

For histopathology, spleen and other tissues from xenograft mice were collected at the days indicated, dipped in 4% formalin-fixed overnight, paraffin embedded, 4–5 μm-thick sections obtained by microtome, and then stained with hematoxylin and eosin (H&E). Images were captured with a 10x Plan-Neofluar NA 0.3 objective and processed with software. For IHC, tissue sections obtained as above were deparaffinized and rehydrated for antigen retrieval, blocked with 10% goat serum in PBS and stained with primary labelled antibodies according to standard procedures. Then, secondary antibodies were used, and the binding was measured with the VIEW DAB Detection Kit.

**Statistical analysis**

Prism 8.0 (GraphPad Software) was used for data analysis. t-test or Two-way analysis of variance (ANOVA) was used to determine the significance of the differences between means in experiments. Survival curves were generated using the Kaplan-Meier method. p<0.05 was considered statistically significant.

Antibodies Table

| Target | Pathway | Clone | Supplier |
| --- | --- | --- | --- |
| CD3 | PE | UCHT1 | BioLegend |
| CD3 | FITC | UCHT1 | Biolegend |
| CD3 | APC | UCHT1 | Biolegend |
| CD4 | APC | A161A1 | Biolegend |
| CD4 | FITC | A161A1 | Biolegend |
| CD5 | APC | UCHT2 | Biolegend |
| CD7 | BV421 | M-T701 | BD |
| CD8 | BV421 | SK1 | Biolegend |
| CD11C | APC | B-ly6 | Biolegend |
| CD14 | PE | 63D3 | Biolegend |
| CD19 | APC | HIB19 | Biolegend |
| CD34 | BV421 | 581 | BD |
| CD45 | PE | H130 | Biolegend |
| CD56 | BV421 | HCD56 | Biolegend |
| CD99 | FITC | 3B2/TA8 | Biolegend |
| CD99 | PE | TU12 | BD |
| CD99 | / | 12E7 | Abcam |
| CD99 | / | 1021527 | R&D |
| HLA-DR | PE | L243 | Biolegend |
| Strep-Tag II | FITC | 5A9F9 | GenScript |
| 7-AAD | PerCP | / | Biolegend |
| Goat Anti-Mouse IgG (H+L) | FITC |  | Jackson |
